# Spatial distribution of trace elements in surface sediments of Hooghly (Ganges) river estuary in West Bengal, India

**DOI:** 10.1007/s11356-021-15918-8

**Published:** 2021-08-31

**Authors:** Marco Trifuoggi, Luciano Ferrara, Maria Toscanesi, Priyanka Mondal, Jonathan Muthuswamy Ponniah, Santosh Kumar Sarkar, Michele Arienzo

**Affiliations:** 1 Dipartimento di Scienze Chimiche, Università degli Studi di Napoli Federico II, Complesso Universitario di Monte Sant’Angelo, via Cintia 26, 80126 Naples, Italy; 2 Department of Marine Science, University of Calcutta, 35 Ballygunge Circular Road, Calcutta, 700019 India; 3 Centro Interdisciplinario de Investigaciones y Estudios sobre Medio Ambiente y Desarrollo, Instituto Politécnico Nacional, Calle 30 de Junio de 1520, Barrio la Laguna Ticomán, C.P. 07340, Del. Gustavo A. Madero, Ciudad de México, Mexico; 4 Dipartimento di Scienze della Terra, dell’Ambiente e delle Risorse, Università degli Studi di Napoli Federico II, Complesso Universitario di Monte Sant’Angelo, via Cintia 26, 80126 Naples, Italy

**Keywords:** Hooghly estuary, Trace elements, Sediment pollution, Multivariate analysis, Pollution index factors, Ecological risk assessment

## Abstract

The spatial distribution of trace elements in surface sediments of the Hooghly estuary was studied over the monsoons in 2014–2017. As, Cd, Ni, Pb and U were two- to sixteen-fold the crust means with increasing levels toward the estuary, with Ni peak during the post-monsoon. Pearson’s correlation matrix, cluster analysis, enrichment factors and pollution index revealed the anthropic source and association of trace elements with Fe, Mn and Al and of Pb with U. Geoaccumulation index revealed for Ni an extremely contaminated situation at the estuary water during monsoon and for Cd a heavily contaminated situation at freshwater location. The potential contamination index was >6; thus, sediments were very severely contaminated by As, Cd and Ni with worst situation for As and Cd at fresh and brackish water and during post-monsoon. The overall ecological risk was severe, 300≤RI<600 at all sites and seasons, especially after the monsoon, at fluvial and brackish locations.

## Introduction

Trace elements, TEs, concentrations in sediments of fluvial and estuarine environments are affected by input coming from discharge of industrial and urban sewage or by atmospheric deposition in the catchment. The Ganges, locally called Ganga, is a large river on the Indian subcontinent that crosses the plains of northern India and Bangladesh. It has a length of 2510 km, and its sources are located on the Gangotri glacier in the Indian state of Uttarakhand in the central Himalayas. It flows into the Bay of Bengal with a large delta in the Sundarbans region. Together with its tributaries, it drains a catchment area that covers about one million km^2^, supporting one of the most densely populated regions of the planet Earth (Sarkar et al. 2017). Almost half of India’s population lives in a third of the country’s territory, within the Ganges Plain (Indo-Ganges). The river basin of Ganga and its estuary can be considered an ideal site for studying the influence of anthropic pressure on the flows of TEs. The Ganges splits into the Padma and the Hooghly River, HR, near Murshidabad district of West Bengal. The Padma flows eastward into Bangladesh, whereas the Hooghly flows south through West Bengal. The catchment area of HR and its estuary, HRE, are highly urbanized, with commercial, light industrial and domestic land use areas. Details of the main features of anthropic pressure on this estuary are reported by a report of state on environment of the West Bengal Pollution Control Board, WBPCB (2009), and by the Central Pollution Control Board, CPCB (2013). This latter survey reveals that in West Bengal, there is a large presence of chemical industries like petrochemical, fertilizer and textile plants and pulp paper mills beside hospital discharging huge volumes of untreated wastes into the river water. The literature reports several studies dealing with the level of pollution by TEs of HR sediments: Ghosh et al. (1983); Chakraborty and Gupta (2003); Dutta et al. (2005); Kar et al. (2008); Jonathan et al. (2010); Paul and Sinha (2013); Sarkar et al. (2017); Paul (2017); and Mondal et al. (2018a, 2018b, 2020).

One of the key environmental factors affecting the destiny of TEs especially in complex morphogeological context like the HR is represented by the climatic conditions: this area is dominated by a sub-tropical climate with cyclic successions of three distinct seasons, a pre-monsoon season extending from March to June, a monsoon season from July to October, and a post-monsoon season from November to February. The monsoon season is dominated by heavy precipitation, ~ 70–80% of the total precipitation and with mean rainfall of ~1700 mm (Rakshit et al. 2014). These intense raining events produce up to ~3000±1000 m^3^/s of mixed volumes of water and sediment to be washed off from the land and then, in the subsequent seasons, i.e., pre-monsoon, decreases up to ~1000±80 m^3^/s (Ray et al. 2015). This seasonal and intense variation of the climatic conditions adds to tide semidiurnal.

Thus, the high variation of the climatic conditions in a very short time span, typical of the monsoon season, greatly affects the composition and levels of TEs in sediments due to large introduction of terrigenous materials and mixing and transport effects on intertidal sediments and sudden changes of sea currents induced by monsoon winds and storms. The mean rate of sediment accumulation in HRE is elevated and ranges from 3.0 to 4.8 mm year^−1^ (Banerjee et al. 2012). The massive input of sediments from anthropic pressure and natural events significantly affects TEs sediment binding. Another factor that influences the interaction of TEs with sediments is the salinity gradient. The consistent input of organic matter, clay and sulphide contents in the first tract of the river can increase TEs enrichment of intertidal sediments, whereas higher salinity conditions in the lower river stretch can decrease sorption (Du Laing et al. 2008). Some authors report, for instance, the chemical shifting of Cd(II) in solution from Cd^2+^_aq_ to CdCl^+^, CdCl_2_, CdCl_3_^−^ and CdCl_4_^2−^ forms with increasing salinity (Battaglini et al. 1993; Helmke 1999). Besides the complexation action of anions, there is also another route through which salinity can alter TEs sorption and occurs when Ca^2+^ and Mg^2+^ compete for metals for sorption sites (Paalman et al. 1994) as is often the case of Zn and Cd (Millward and Liu 2003).

The current study, carried out over 2014–2017, determined the effect of the anthropic pressure and the water physical chemical features on TEs levels in three distinct specific areas of HRE over the monsoons: an upstream freshwater zone, a downstream brackish water zone and an estuarine saline water zone. For each year, season and zone, classical statistics, multivariate statistical analyses and pollution factor methods were applied, as well the environmental risk, the potential ecological risk, ${E}_r^i$, and comprehensive ecological hazard indices, RI.

## Materials and methods

### Sampling sites

**Fig. 1:**
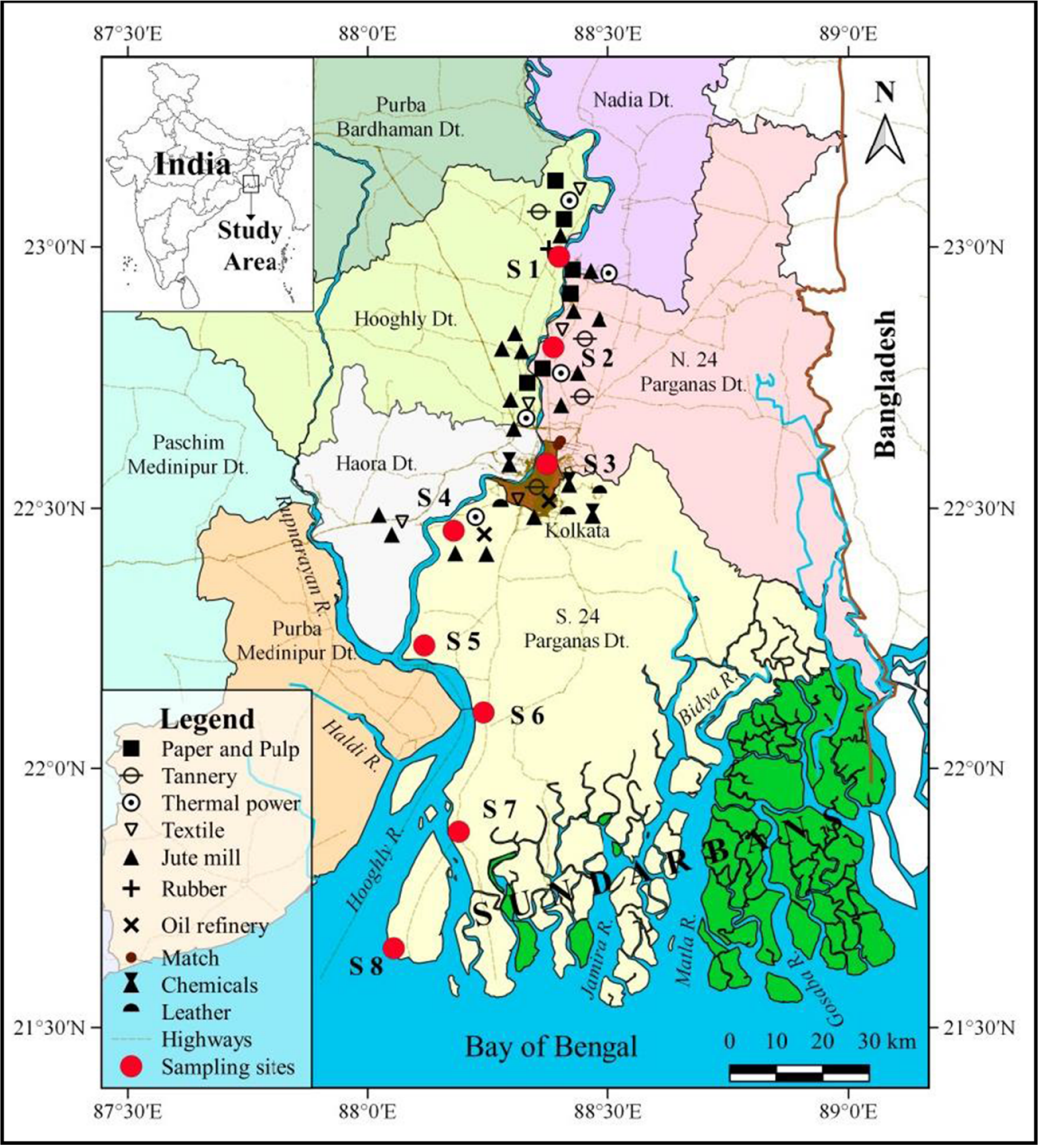
Map showing the location of eight sampling sites (S1 to S8) along the Hooghly river estuary. The intricate river network and position of industries are also shown

Eight sites, namely, Tribeni, S1; Barrackpore, S2; Babughat, S3; Budge Budge, S4; Nurpur, S5; Diamond Harbour, S6; Lot 8, S7; and Gangasagar, S8 (Figure 1), were sampled over the seasons along the ~175-km-long tidal stretch of the HRE from November 2014 to May 2017 for a total of 176 specimens. The chosen sites present different environmental conditions, where S1–S4 are fluvial sites with no tidal influence, S5–S6 show brackish water, while S7–S8 are typically estuarine stations. S8 is situated at the confluence of river Hooghly and Bay of Bengal, while S1 is in the upstream region.

### Collection and pre-treatment of sediments

Water salinity was determined by conductivity and temperature measurement with a resolution of 0.01 psu according to the standard water analysis method (APAT-IRSA CNR 2003). Surface sediments were taken by a Van Veen grab and placed in plastic bags using plastic spatula in triplicate from the intertidal regions during ebb tide covering pre-monsoon, monsoon, and post-monsoon season (Arienzo et al. 2020a, b). Samples were kept in acid-rinsed polyethylene bags and temporarily stored in a cooler box with ice packs at 4°C. The sediments were stored at −20°C until further analyses. They were oven-dried at 80°C to constant weight and gently grounded and crushed (Trifuoggi et al. 2017; Sarkar et al. 2017; Mondal et al. 2018a).

### Analysis of sediments

Dry sediment of ≤2 mm was analysed for pH, organic carbon (C_org_), carbonate, granulometric parameters and TEs, Al, As, Cd, Co, Cr, Cu, Fe, Hg, Mn, Ni, Pb, U and Zn as described in previous works (Trifuoggi et al. 2017; Sarkar et al. 2017; Mondal et al. 2018a). For TEs, ~ 0.5 g was digested with 10 ml of a HCl-HNO_3_-H_2_O_2_ mixture, 6:3:1, and assisted by microwave (Mars-CEM, US). After cooling, the samples were filtered and taken to final volume of 50 ml with ultrapure water. Mineralized samples were analysed by ICP-MS (Aurora M90 Bruker, USA) in triplicate. The detection limit, LOD, and limit of quantification, LOQ, were calculated using the method of blank variability for each investigated element. The calculated average values of LOD and LOQ were 0.06 and 0.16 μg/kg, respectively. The accuracy, precision and recovery were evaluated using certified reference materials CR-CRM667, European Commission, Joint Research Centre, Belgium, and by participation to inter-laboratory circuits. The percentage recovery was 60–120% and the RSD, *n*=3, 2–15%. Statistical analysis consisted of skewness and kurtosis statistical tests (Zhang et al. 2009), Pearson’s correlation matrix, a principal component analysis (PCA) and a hierarchical cluster analysis (HCA) and was performed by Statistica v.5 (StatSoft Inc., Tulsa, OK, USA).

## Results and discussion

### Properties of sediments

The geochemical characteristics of sediments are summarized in Table 1. Data are splitted for salinity area of the studied sites: the fluvial sites S1–S4 with a salinity of 0.13–0.3 psu, the brackish sites S5 and S6 with a salinity of 1.1–6.0 psu and the estuarine stations S7 and S8 with a salinity of 7.5–22.5 psu. Overall, the mean pH values of the brackish, 7.55–7.59, and estuary areas, 7.44–7.46, are slightly basic and higher than those of the freshwater location, 7.26–7.41, with maximum slightly basic peaks of 8.11 at S5 and minimum slightly acidic values at S3, 6.77, and S4, 6.85. Values reveal a certain increasing pH trend toward the outer part of the estuary due to marine influence. The C_org_, expressed as %, fluctuated among the sediment samples with no regular distribution pattern. Very low content of C_org_ was observed in the intertidal sediments of HRE, with mean values of 0.34–0.51% in the fluvial area, 0.39–0.47% in the brackish area and 0.42–0.43 % in the estuary. These values were lower than those found in sediments from other Indian coastal areas, such as Gulf of Mannar (Jonathan and Ram Mohan 2003), cochin (Sunil Kumar 1996) and Muthupet mangroves (Janaki-Raman et al. 2007), and match with those reported by Subba Rao (1960), reporting very low organic carbon values in the shelf sediments of the east coast of India (Krishna and Godavari basins) made of very fine grains of clay and silt. Peaks of C_org_ % were determined at S3, 1.14; S4, 0.75; and S6, 0.84. The lowest concentration of organic carbon in the outer stations is due to a large difference in environmental conditions regarding the rate of sediment deposition, tidal effects, microbial degradation (Antizar-Ladislao et al. 2015) and sediment sorption capacity for organic compounds (Sarkar et al. 2004; Chatterjee et al. 2007).

**Table 1:** Sampling location associated to mean values of salinity, pH, C_org_, CaCO_3_ and grain size of sediments from HRE

Zones	Sampling sites	Latitude N	Longitude E	pH	C_org_ (%)	CaCO_3_ (%)	Sand (%)	Silt (%)	Clay (%)	Class*
Freshwater ~90 km	S1 Tribeni	22°59′25″	88°24′12″	7.27 (6.93–7.90)	0.34 (0.15–0.72)	12.46 (9.09–15.60)	19.49 (11.22-32.08)	30.80 (8.94–50.6)	49.71 (36.91–75.72)	Clay loam
	S2 Barrackpore	22°45′51″	88°20′40″	7.41 (7.05–7.73)	0.42 (0.24–0.63)	13.43 (10.04–16.33)	15.84 (2.34-30.06)	35.19 (17.15–62.18)	48.97 (14.89–70.54)	Clay loam
	S3 Babughat	22°49′32″	88°21′39″	7.39 (6.77–7.76)	0.51 (0.15–1.14)	11.48 (4.4–14.69)	16.78 (3.35-28.89)	34.96 (23.23–55.43)	48.52 (35.42–64.09)	Clay loam
	S4 Budge Budge	22°33′58″	88°11′16″	7.26 (6.85–7.53	0.51 (0.18–0.75)	11.4 (8.33–13.43)	14.96 (4.96-22.33)	33.43 (23.7–62.33)	51.60 (29.79–71.34)	Clay loam
Brackish ~23 km	S5 Nurpur	22°12′40″	88°04′16″	7.59 (6.80–8.11)	0.39 (0.15–0.65)	11.14 (7.20–13.89)	7.45 (2.15-13.45)	32.93 (24.26–61.70)	59.62 (36.15–68.52)	Clay loam
	S6 Diamond Harbour	22°11′13″	88°11′24″	7.55 (7.16–7.94)	0.47 (0.12–0.84)	11.20 (6.34–14.74)	8.73 (4.36-13.67)	32.85 (24.98–63.29)	58.43 (31.68–66.01)	Clay loam
Estuarine ~63 km .	S7 Lot 8	22°52′29″	88°10′09″	7.46 (7.03–7.83)	0.42 (018–0.76)	11.71 (1.98–15.54)	10.45 (0.9-20.09)	32.22 (7.25–48.76)	58.33 (31.15–87.55)	Clay loam
	S8 Gangasagar	22°38′24″	88°04′46″	7.44 (7.13–7.91)	0.43 (0.15–0.82)	9.98 (6.37–15.54)	34.61 (17.29-65.42)	28.43 (17.00–40.38)	36.96 (16.98–51.6)	Clay loam-sand

*Classification of sediment grain size (Folk and Ward 1957)

Minimum and maximum value of each parameter is given in parenthesis

The content of CaCO_3_ was generally high and with a clear decreasing trend from the inner part of the estuary toward the open sea, with mean values varying from 12.46% at S1 to 9.98% at S8. This is linked to multiple causes like the greater fluvial content of carbonate, in the form of carbonate deposits and flood-related deposit common in tropical settings (Carthew et al. 2003), as well as to greater transport and mixing, especially during the monsoon season, of terrigenous fraction from continental sediments. We also estimated the variation of carbonates at each site over the season, and we observed significant increasing levels from monsoon to pre-monsoon season up to 4.7% at S7. The grain size parameters show how the surface sediments of HRE can be classified into two primary textural groups: clay loamy and clay loamy sandy according to the classification of Folk and Ward (1957). Clay is very abundant, with a mean range of 37.0–59.6% and a maximum in the estuarine site S7, 87.55%, with a clear decreasing trend from freshwater to estuary zone due to progressive sedimentation of finest particles. A case apart is represented by S8 where clay reaches its minimum value, 37%, located at the confluence of HR and Bay of Bengal, endorsing high-energy zone. Silt contents are rather homogenous with a narrow range of mean variation, 28.43–35.19%, whereas sand shows a higher variability with a mean range of 14.94–15.84% in the fluvial, 7.45–8.73 % in the brackish and 10.45–34.6% in the estuary. The dominance of finer sediment, clay and silt, indicates a weak hydrodynamic condition of the estuary and is also an indication of freshwater input with finer particles that settle to the bottom when current and winds reduce. The mean diameters of the surface sediments vary generally from 3.4 to 4.86 φ and reach their peaks at the mid to lower stretch of the estuary. The coarsest sediments (<3.5 φ) occur at S8 at the mouth of the estuary. The sorting coefficients of the surface sediments varied from 0.575 to 1.03 φ, indicating moderately well sorting in the study region which might be attributed to the relatively better distribution of the finer sediments. Size distributions in the study area were fine skewed, with values of skewness varying from 0.194 φ at S_3_ to 0.538 φ at S_6_. The main factors affecting the spatial distribution of grain size parameters are sediment sources and characteristics, hydrodynamic conditions and topography features (Liang et al. 2020). Kurtosis analyses show that all samples, except S7, are leptokurtic in nature.

### Distribution of trace elements in the sediments and assessment of contamination

Table 2 reports the range of variation of the trace elements over 2014–2017 for the three HRE salinity areas and along each season. Data were compared with the upper chemical composition of the Earth’s continental crust level, ECCL (Taylor and McLennan 1985), used as background, since there are no data on background concentrations for the studied sediments of the region. TEs along the studied period and according to the mean concentration are scaled with the following order: Fe>Al>Mn>Ni>Cr>Zn>Pb>Cu>Co>As>U>Cd>Hg. Most of them were below the mean level of the upper continental crust except As, Cd, Ni, Pb and U which were about four, six, nine, two and twofold the crust means.

**Table 2:** Mean, minimum and maximum concentration of TEs in sediment of fresh, brackish and estuary waters in 2014–2017 and seasons

Estuary portion	Al	As	Cd	Co	Cr	Cu	Fe	Hg	Mn	Ni	Pb	U	Zn
2014–2017													
Mean ±SD	4.86±1.03	6.63±0.65	0.57±0.12	11.4±0.8	57.8±5.1	28.3±2.1	35.3±19.0	0.08±0.02	593±40	189±27	32.4±0.54	4.30±0.09	57.3±2.09
Freshwater	62.6	6.71	0.70	10.3	51.1	26.2	33.3	0.08	536	158	31.7	4.4	54.8
	5.07–8.44	3.88-8.99	0.55-0.93	7.3–13.3	38.7–65.4	19.2–34.6	30.5–37.5	0.06–0.10	486–617	119–184	26.4–37.1	3.78–5.13	37.3–75.7
Brackish water	4.54	5.80	0.61	11.8	58.8	27.6	34.8	0.09	629	185	32.6	4.21	57.1
	3.84–5.10	4.81-7.12	0.55-0.93	10.2–14.6	51.7–71.9	21.8–36.2	32.6–38.8	0.05–012	576–702	164–194	30.1–36.0	3.92–4.35	47.6–71.0
Estuary water	3.78	7.41	0.42	12.2	63.5	31.1	37.9	0.08	613	224	33.0	4.42	59.9
	3.40–4.48	6.61-8.75	0.34-0.50	9.1–15.5	50.8–79.7	22.4–40.1	32.6–45.0	0.06–0.09	516–747	194–248	28.7–37.5	3.85–4.76	43.1–80.6
Pre-monsoon													
Freshwater	5.07	7.26	0.67	9.69	49.1	24.9	31.8	0.08	486	171	31.6	4.43	51.4
	3.06–11.5	1.10–27.0	0.20–2.84	2.8–28.2	10.7–162	5.5–86.0	17.7–56.1	0.03–0.23	320–851	48.0–276	18.2–58.0	2.14–9.50	10.69–193
Brackish water	3.84	5.38	0.55	10.7	52.7	24.7	33.1	0.08	576	198	31.6	3.92	52.8
	2.66–6.12	1.19–24.2	0.13–2.08	2.8–31.1	11.1–165	4.6–98.0	19.2–62.5	0.04–0.24	327–1191	86.0–321	21.4–60.0	2.50–7.1	9.74–176
Estuary water	3.40	6.61	0.41	12.1	59.9	30.9	35.9	0.07	576	229	32.8	4.5	56.1
	2.37–5.60	1.33–19.0	0.13–1.56	3.4–42.1	15.5–202	6.7–109	25.1–60.3	0.03–0.17	334–966	72.0–413	20.8–66.0	2.43–7.1	13.01–183
Monsoon													
Freshwater	5.25	3.88	0.55	7.79	38.7	19.2	30.5	0.06	504	184	26.4	3.78	37.3
	2.97–11.92	1.47–17.9	0.15–1.78	3.3–26.4	15.7–126	7.1–73.0	21.0–54.0	0.04–0.15	357–1041	53.0–272	18.8–51.0	2.43–7.5	11.95–146
Brackish water	5.10	4.81	0.63	10.2	51.7	21.8	32.6	0.05	610	194	30.1	4.35	47.6
	2.77–15.45	1.70–16.4	0.21–3.12	3.0–47.4	16.6–244	6.0–102	21.8–91.4	0.03–0.11	357–1927	74.0–257	16.4–99.0	2.36–16.5	10.96–239
Estuary water	3.47	6.81	0.34	9.1	50.8	22.4	32.7	0.06	516	248	28.7	3.85	43.1
	2.43–5.83	1.76–28.3	0.14–1.26	3.9–30.0	22.2–165	9.4–83.0	20.8–61.1	0.03–0.14	377–908	87–365	18.0–60.0	2.50–6.20	15.40–164
Post-monsoon													
Freshwater	8.44	8.99	0.93	13.3	65.4	34.6	37.5	0.10	617	119	37.1	5.13	75.7
	3.52–20.76	0.68–34.4	0.20–3.38	1.8–33.4	7.8–176	3.4–92.0	21.4–63.2	0.04–0.09	334–747	50.0–194	20.6–37.5	2.33–4.76	6.24–80.6
Brackish water	4.68	7.12	0.69	14.6	71.9	36.2	38.8	0.12	702	164	36.0	4.32	71.0
	2.70–8.05	1.26–17.0	0.20–1.90	3.5–38.9	15.9–183	7.3–95.0	13.0–60.8	0.05–0.31	361–1122	52.0–288	22.5–65.0	2.59–8.5	12.42–192
Estuary water	4.48	8.75	0.50	15.5	79.7	40.1	45.0	0.09	747	194	37.5	4.76	80.6
	2.86–8.00	1.90–22.2	0.14–1.28	4.8–33.6	22.4–166	10.1–85.0	25.6–69.3	0.04–0.26	388–1182	83.0–344	22.1–68.0	2.83–8.3	17.97–168
Upper continental crust	80.4	1.5	0.098	10.0	35.0	25.0	35.0	0.05	600	20.0	20.0	2.80	71.0

The concentrations are in mg/kg except Fe and Al, g/kg

The mean spatial distribution revealed that while Al significantly decreased from the fluvial area toward the mouth of the estuary, 62.6 vs. 37.8 g/kg, the levels of Fe and Mn tended to increase by ~10–20%. In parallel to this trend, the concentrations of all the other elements, except Cd which decreased by 40%, increased, as in the case of Ni, up to 29.5% from the freshwater area to the estuary location. TEs, in fact, tend to bound to amorphous and well-crystallized iron and manganese oxyhydroxides (Yin et al. 2016) and under these forms are transported down the studied estuary. Only the mean levels of Hg and U remained nearly unvaried. This trend remains approximately the same over the three different seasons, with a peak of As accumulation during the monsoon in estuary water, 3.88 vs. 6.81, corresponding to an increase of ~ 75.52% and of Ni in the post-monsoon season, 119 vs. 194, i.e. ~63%. By contrast, Ni in the post-monsoon season seems to be permanently washed off up to ~15–30% in the fluvial and brackish area. The lowest mean concentrations of Co, Ni, Cu, Zn and As were encountered at the sampling site Babughat (S3) during post-monsoon season (November 2016), while the maximum concentration of Mn, Fe, Co and Zn was recorded at Diamond Harbour (S6) during monsoon season (August 2015).

Table 3 displays the comparison of the mean TEs concentrations over 2014–2017 in superficial sediments with those from the main water courses and the major eastern, western and southern estuary of India and other world spots as South Yellow Sea and Mediterranean coastal areas. On the overall, our results do not result so alarming being the TEs levels of the same order of magnitude or even lower than those of the other considered locations. However, this is not the case of As which is up to tenfold higher than the Ganges (Banerjee et al. 2012) and Krishna estuary (Ramesh et al. 1999) and Tapti and Cochin site (Sharma and Subramanian 2010; Balachandran et al. 2006). The reason for the presence of arsenic is all probably geological as reported by Chakraborty et al. (2018). Also, Ni is rather worrying since its mean level, 190 mg/kg, is fourfold of those from other sites of India and ~ thirteenfold higher of those of the Ganges estuary (Banerjee et al. 2012). All investigated TEs fall within the range of the Mediterranean coastal area (UNEP 1996).

**Table 3:** Comparison of TEs concentrations in sediments with other fluvial and coastal regions of India and other parts of the world

River/estuary region	Al	As	Cd	Co	Cr	Cu	Fe	Hg	Mn	Ni	Pb	Zn	References
Hooghly river estuary													
Freshwater	62.6	6.71	0.70	10.3	51.1	26.2	333	0.08	536	158	31.7	54.8	This study
Brackish water	45.4	5.80	0.61	11.8	58.8	27.6	348	0.09	629	185	32.6	57.1	“
Estuary water	37.8	7.41	0.42	12.2	63.5	31.1	379	0.08	613	224	33.0	59.9	“
Rivers													
Old Brahmaputra	90.0		0.48	4.10	6.60	6.20		0.001	126	12.8	7.60	52.7	Bhuyan et al. (2019)
Shitalakhya	304.3			13.37	74.82	143.7						200	Islam et al. (2016)
Meghna			0.23		31.74				442.6	76.1	9.47	79.0	Hassan et al. (2015)
Buriganga			1.50		173.4	344			4036	153	31.4	481	Mohiuddin et al. (2015)
Karatoa			1.20		109					95	58.0	.	Islam et al. (2015)
Bangshi			0.61		98.1				483	25.67	60.0	117	Rahman et al. (2014)
Karnofuly			0.24		0.76	1.22			15.3		4.96	16.3	Islam et al. (2013)
Turag			1.4		0.44	1.60					1.64	1.08	Banu et al. (2013)
						Estuary east coast							
Cauvery					49.5	29.5	523		160	13.5	8.5	30	Dhanakumar et al. (2013)
Ganges		0.08	2.01	18.23	40.1	21.6	286		502	34	23.4	53	Banerjee et al. (2012)
Godavary			24.8	25.5	71.2	103				63.8	424	3867	Krupadam et al. (2007)
Krishna		0.14	0.99	37.8	148					95	4.81	171	Ramesh et al. (1999)
						Estuary west coast							
Tapti		1.70	0.50	27	212	326	911		1498	205	25	216	Sharma and Subramanian (2010)
Narmada		1.60	1.10	25.9	199	188	896		1214	203	13.9	196	Sharma and Subramanian (2010)
Ulhas				64.1	496	130	780		1151	98		217	Rokade (2009)
Cochin			5.91	18.3	82.3	30.8	447		229	53.7	38.7	562	Balachandran et al. (2006)
						South east coast							
Bay of Bengal			6.58	8.10	194	506	272		373	38.6	32.6	126	Muthu Raj and Jayaprakash (2008)
						Other sites							
South Yellow Sea			0.30			16.9					17.8	93.7	Hu et al. (2013)
Mediterranean coastal areas			0.02–64			0.5–1890		0.05–0.10			3-3300	1.7–6200	UNEP (1996)

The concentrations are in mg/kg except Fe and Al, g/kg

### Correlation analysis

Table 4 reports the output of the Pearson correlation matrix (CM) applied to the data Al, As, Cd, Cr, Cu, Fe, Hg, Mn, Ni, Pb, U, Zn, pH, C_org_ CaCO_3_, sand, silt and clay for freshwater zone. The major outputs of the Pearson’s data reveal the absence of any correlation of TEs with clay and silt along the entire stretch of the river examined. Most of TEs, except for Hg, appear significantly correlated, *p*<0.05, *r* ranges 0.70–0.99, highlighting a common source. They are also correlated with Mn, *r* ranges 0.70–0.92, meaning an evident association with this element. This, together with the already observed spatial distribution of Fe and Mn along the estuary, supports the hypothesis that TEs are bound to amorphous and/or crystallized iron and manganese oxyhydroxides. However, Ni differs from this behaviour, for which we observe an important negative correlation with Co, Cr, Cu and Zn, r ~−075, indicating a different provenience of the element. Another interesting issue regards the couple Pb-U which were highly significantly correlated, *r* of 0.89. This finding could be likely linked to the massive presence of the fertilizer industry in the first stretch of the estuary and the use of phosphorites (phosphoric mineral), which, depending on their origin, may have high concentrations of radioactive elements of the natural series uranium, ^238^U, besides thorium, ^232^Th; potassium, ^40^K; and ^210^Pb. In phosphoric acid production, the radioactive equilibrium in the phosphate rock is disrupted, with ^238^U and ^232^Th and ^210^Pb appearing primarily in the phosphoric acid, while the ^226^Ra and ^210^Po becomes associated with the gypsum waste product (Paul and Pillai 1990). The concentrations of uranium in phosphorite rocks are generally between 30 and 260 mg/kg and are higher than the average uranium ECCL content of 2.80 mg/kg (Taylor and McLennan 1985); see Table 1.

**Table 4:** Correlation coefficients among TEs, pH, C_org_, CaCO_3_, sand, silt and clay for freshwater sites

	Al	As	Cd	Co	Cr	Cu	Fe	Hg	Mn	Ni	Pb	U	Zn	pH	C_org_	CaCO_3_	Sand	Silt	Clay
Al	1.00																		
As	0.27	1.00																	
Cd	0..35	0.64	1.00																
Co	0.45	**0.91**	0.68	1.00															
Cr	0.44	**0.92**	0.67	**0.99**	1.00														
Cu	0.42	**0.91**	0.65	**0.98**	**.98**	1.00													
Fe	0.55	0.69	0.54	**0.83**	**0.81**	**0.81**	1.00												
Hg	0.15	0.39	0.30	0.39	0.36	0.41	0.40	1.00											
Mn	0.60	**0.70**	0.60	**0.85**	**0.84**	**0.83**	**0.92**	0.40	1.00										
Ni	−0.58	−0.67	−0.54	−**0.74**	−**0.75**	−**0.73**	−0.59	−0.28	−0.68	1.00									
Pb	0.34	0.48	0.48	0.54	0.54	0.55	0.46	0.30	0.43	−0.42	1.00								
U	0.41	0.56	0.47	0.60	0.61	0.059	0.49	0.20	0.48	−0.50	**0.89**	1.00							
Zn	0.45	**0.92**	**0.70**	**0.99**	**0.99**	**0.99**	**0.81**	0.38	**0.84**	−**0.76**	0.54	0.60	1.00						
pH	−0.12	−0.29	−0.21	−0.34	−0.34	−0.31	−0.26	0.14	−0.29	0.08	−0.01	−0.13	−0.33	1.00					
C_org_	0.09	0.26	0.36	0.23	0.20	0.22	0.16	0.14	0.21	0-.21	0.08	0.10	0.24	−0.11	1.00				
CaCO_3_	0.04	0.16	0.06	0.16	0.14	0.17	0.26	0.09	0.12	0.03	0.08	0.04	0.16	−0.07	0.03	1.00			
Sand	−0.022	−0.48	−0.49	−0.52	−0.50	−0.51	−0.41	−0.39	−0.45	0.28	−0.34	−0.27	−0.50	0.26	-0.26	-0.21	1.00		
Silt	0.28	0.23	0.23	0.28	0.26	0.24	0.38	0.25	0.41	−0.24	0.01	0.09	0.26	−0.28	0.20	-0.05	-0.23	1.00	
Clay	−0.12	0.09	0.11	0.08	0.08	0.11	−0.09	0.02	−0.09	0.04	0.21	0.09	0.09	0.09	-0.02	0.18	-0.44	**-0.78**	1.00

In bold the values statistically significant (*p* < 0.05)

Table 5 displays the Pearson’s CM for brackish water. In this case, there are a larger number of elements, Al, As, Cd, Co, Cr Cu, Fe, Mn and Pb which appear correlated each to other, with *r* of 0.72–0.99. Mn that in freshwater appears correlated only with Co, Cr, Cu, Fe and Zn, *r* of 0.82–0.92, in brackish water correlates with all set of TEs, *r* range of 0.73–0.97, and this seems to match with the already higher levels of this element in this water. It is also interesting to note, besides the negative and significant correlation of Ni, that both Pb and U correlate with all the studied set of TEs, *r* of 0.70–0.96. In the case of estuary water, data not shown, the correlation features return almost identical to those of the freshwater.

**Table 5:** Correlation coefficients among TEs, pH, C_org_, CaCO_3_, sand, silt and clay for brackish water sites

	Al	As	Cd	Co	Cr	Cu	Fe	Hg	Mn	Ni	Pb	U	Zn	pH	C_org_	CaCO_3_	Sand	Silt	Clay
Al	1.00																		
As	0.43	1.00																	
Cd	**0.72**	**0.74**	1.00																
Co	0.65	**0.89**	**0.90**	1.00															
Cr	0.67	**0.90**	**0.90**	**1.00**	1.00														
Cu	0.59	**0.92**	**0.83**	**0.98**	**0.98**	1.00													
Fe	**0.73**	**0.73**	**0.79**	**0.85**	**0.85**	**0.84**	1.00												
Hg	0.11	0.32	0.21	0.36	0.32	0.39	0.33	1.00											
Mn	**0.79**	**0.73**	**0.83**	**0.86**	**0.87**	**0.83**	**0.97**	0.32	1.00										
Ni	−0.54	−**0.74**	−0.69	−**0.76**	−**0.74**	−**0.75**	−**0.72**	−0.41	−**0.73**	1.00									
Pb	**0.70**	**0.81**	**0.87**	**0.95**	**0.95**	**0.92**	**0.80**	0.21	**0.81**	−0.66	1.00								
U	**0.80**	**0.71**	**0.88**	**0.88**	**0.89**	**0.82**	**0.77**	0.12	**0.81**	−0.60	**0.96**	1.00							
Zn	0.64	**0.91**	**0.88**	**0.99**	**0.99**	**0.98**	**0.85**	0.36	**0.87**	−**0.78**	**0.94**	**0.87**	1.00						
pH	−0.16	−0.19	−0.12	−0.21	−0.21	−0.21	−0.15	−0.24	−0.19	0.15	−0.18	−0.19	−0.22	1.00					
C_org_	0.11	0.45	0.19	0.33	0.33	0.40	0.40	0.40	0.34	−0.30	0.28	0.17	0.35	−0.37	1.00				
CaCO_3_	0.01	0.19	0.09	0.19	0.20	0.22	0.22	0.25	0.19	−0.09	0.11	0.06	0.19	0.02	0.14	1.00			
Sand	−0.18	−0.38	−0.30	−0.41	−0.40	−0.44	−0.33	−0.03	−0.35	0.26	−0.35	−0.28	−0.39	−0.13	−0.06	−0.14	1.00		
Silt	0.54	0.46	0.52	0.60	0.59	0.56	0.55	0.21	0.59	−0.40	0.61	0.56	0.58	−0.07	0.05	0.19	−0.51	1.00	
Clay	−0.54	−0.40	−0.49	−0.55	−0.53	−0.48	−0.51	−0.22	−0.56	0.37	−0.57	−0.54	−0.52	0.13	−0.03	−0.17	0.24	−**0.96**	1.00

In bold the values statistically significant (*p* < 0.05)

### Factor analysis

The loading factors, total and cumulative variance generated by the principal component analysis of TEs, C_org_, CaCO_3_, sand, silt and clay are shown in Table 6. For freshwater, two principal components account for 59.4% of total cumulative variance. PC1 explains 48.4% of total variance and is significantly and negatively correlated with As, Cd, Cr, Cu, Fe and Mn and positively correlated with Ni with a moderate positive load. PC2 groups have only two significant loads: one is clay, high positive load of 0.94, and the other is silt, moderate negative significant load of −0.83. In the case of brackish water, PC1 and PC2 explain 59.1% and 9.2% of total cumulative variance with all the elements grouping under component 1 and with positive significant loads. Only Ni displayed a significant negative load of −0.78. The estuary water shows components 1 and 2 explaining 52.8 and 11.7 % of the total variance and with elements grouping all together under PC1 with positive loads except for Ni and high negative load of 0.92.

**Table 6:** Loading factors, total and cumulative variance of TEs, pH, CaCO_3_, sand, silt and clay for fresh, brackish and estuary water

	Freshwater		Brackish water		Estuary water	
	PC1	PC2	PC1	PC2	PC1	PC2
Al	−0.55	−0.23	**0.74**	0.27	**0.71**	0.32
As	−**0.89**	0.08	**0.87**	−0.21	**0.92**	−0.09
Cd	−**0.74**	0.07	**0.90**	0.10	**0.83**	−0.22
Co	−**0.97**	0.02	**0.98**	−0.04	**0.96**	−0.15
Cr	−**0.96**	0.03	**0.98**	−0.03	**0.97**	−0.15
Cu	−**0.96**	0.07	**0.96**	−0.12	**0.96**	−0.15
Fe	−**0.86**	−0.16	**0.90**	−0.05	**0.90**	0.16
Hg	−0.45	0.02	0.36	−0.55	0.42	0.17
Mn	−**0.89**	−0.20	**0.92**	0.01	**0.92**	0.12
Ni	**0.77**	0.10	−**0.78**	0.17	−**0.90**	0.07
Pb	−0.63	0.34	**0.94**	0.09	**0.93**	−0.05
U	−0.68	0.21	**0.90**	0.18	**0.74**	0.06
Zn	−**0.97**	0.04	**0.98**	−0.08	**0.98**	−0.14
pH	0.32	0.24	−0.22	0.49	0.15	0.14
Corg	−0.28	−0.09	0.36	−**0.71**	0.25	0.11
CaCO_3_	−0.17	0.24	0.20	−0.17	0.02	−0.52
Sand	0.57	−0.28	−0.42	−0.29	0.15	**0.92**
Silt	−0.34	−**0.83**	0.68	0.44	0.20	−0.14
Clay	−0.05	**0.94**	−0.63	−0.40	−0.25	−**0.85**
Initial eigenvalue	9.20	2.07	11.23	1.74	10.04	12.24
% total variance	48.4	10.9	59.1	9.2	52.8	11.7
% cumulative variance	48.4	59.4	59.1	68.3	52.8	64.5

In bold the significant loads, *p*<0.05

These results confirm those from Pearson’s CM showing an evident association of most TEs with major elements Fe and Mn, likely in the form of amorphous or crystalline iron and manganese oxides. Results also highlight the different sources of Ni and the scant role of the fine clay and silt fractions from terrestrial debris.

### Hierarchical cluster analysis

**Fig. 2:**
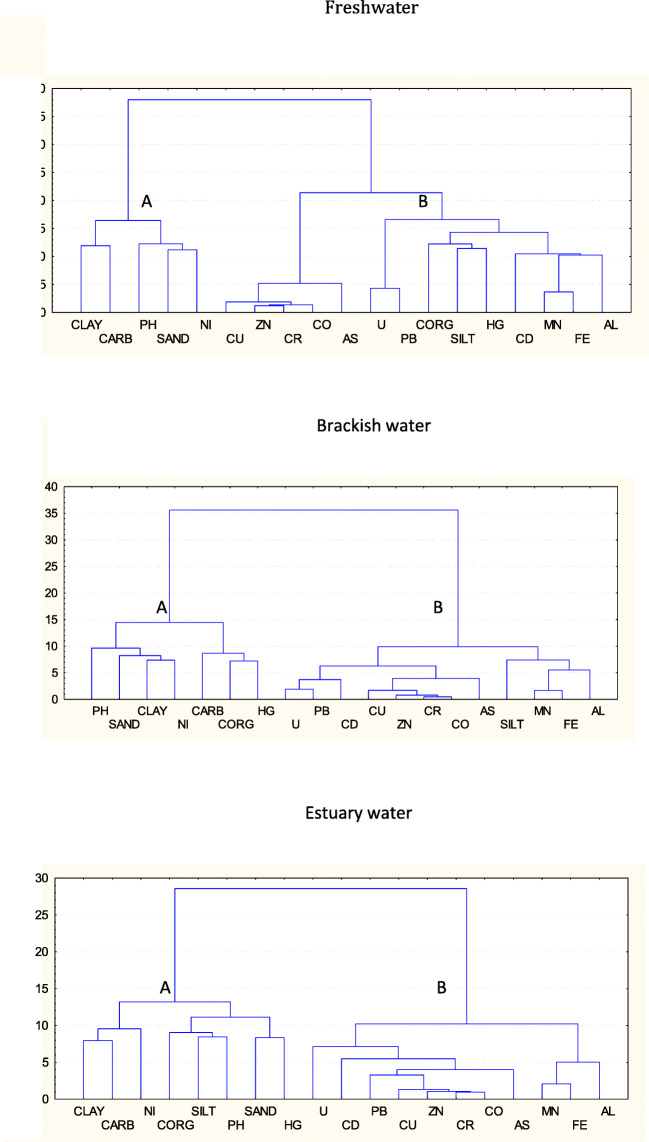
Output of hierarchical cluster analysis (HCA). Letters indicate clusters

The hierarchical cluster analysis (HCA), Fig. 2, was performed by the criteria of Ward (Lebart et al. 1984). The diagrams allow us to clearly recognize for all the three water systems two main clusters, A and B, that are very distinct at high hierarchical level. This means that the variables split into two main groups which clearly characterize the estuary: cluster A including clay, CaCO_3_, pH, sand, clay and Ni and cluster B including all elements, with very short distance between Al, Fe and Mn from one side and narrow distance between Pb and U, once again confirming the role of the major elements, Al, Fe and Mn in enriching and transporting elements along the estuary.

### Index of geoaccumulation (Igeo)

The degree of contamination was assessed by the geoaccumulation index (Igeo); enrichment factor, EF; contamination factor (CF); contamination degree, Cd; modified contamination degree, mCD; pollution load index, PLI; and potential contamination index, Cp (Muller 1969; Bryan and Langston 1992; Ravichandran et al. 1995; Buccolieri et al. 2006; Vaezi et al. 2016; Barbieri 2016; Arienzo et al. 2020a, 2020b) (Tables 7, 8, 9).

**Table 7:** Geoaccumulation index (Igeo) and enrichment factor (EF)

	Al	As	Cd	Co	Cr	Cu	Hg	Mn	Ni	Pb	U	Zn
Freshwater												
Igeo	−2.582	2.457	3.380	0.511	0.995	0.473	1.296	0.333	3.174	1.295	1.297	−0.051
EF	0.080	4.013	7.257	0.943	1.337	0.943	1.580	0.940	9.89	1.720	1.707	0.677
Brackish water												
Igeo	−3.740	1.926	2.677	0.256	0.740	0.094	0.855	0.481	3.648	1.155	0.995	−0.504
EF	0.05	3.04	4.87	0.90	1.29	0.85	1.31	0.96	11.48	1.55	1.39	0.57
Estuary water												
Igeo	−3.954	2.194	2.201	0.406	0.963	0.317	0.785	0.505	3.872	1.189	1.145	−0.311
EF	0.04	3.88	3.51	1.00	1.49	0.99	1.24	0.95	12.37	1.71	1.52	0.68

**Table 8:** Contamination factor (Cf), modified contamination degree (mCD) and pollution load index (PLI)

Cf													mCd	PLI
Al	As	Cd	Co	Cr	Cu	Fe	Hg	Mn	Ni	Pb	U	Zn		
Freshwater														
0.08	4.47	7.31	1.03	1.46	1.05	0.95	1.60	0.89	7.90	1.59	1.59	0.77	2.36	1.39
Brackish water														
0.06	3.85	6.36	1.18	1.68	1.10	1.00	1.67	1.05	9.27	1.63	1.50	0.80	2.39	1.41
Estuary water														
0.05	4.93	4.25	1.22	1.81	1.25	1.08	1.47	1.02	11.18	1.65	1.56	0.85	2.49	1.43

**Table 9:** Potential contamination index (Cp)

Al	As	Cd	Co	Cr	Cu	Fe	Hg	Mn	Ni	Pb	U	Zn
Freshwater												
0.18	17.6	27.2	2.93	4.42	3.35	1.65	3.1	1.46	12.4	2.44	2.59	1.97
Brackish water												
0.12	12.8	24.1	3.91	5.64	3.93	2.05	4.4	2.35	14.4	3.73	3.82	2.85
Estuary water												
0.1	15.5	13.9	3.52	5.07	3.69	1.82	3.8	1.70	18.7	3.23	2.57	2.42

Igeo was calculated from the ECCL (Taylor and McLennan 1985) used as background, and results interpreted according to the seven grades proposed by Müller (1981). Table 7 shows how a heavily to extremely contaminated class was individuated for Ni, 3<Igeo<4, at all sites, with an extremely contaminated situation at the estuary, ~4. The second worrying situation was found for Cd in correspondence of the freshwater location, with mean Igeo values of 3.38, falling in heavily contaminated class and with no significant differences among seasons. Whatever was the season, brackish and estuary water showed lower Igeo mean values, 2.68 and 2.20, respectively, falling in the class of moderately contaminated-heavily contaminated class, 2<Igeo<3. A similar trend was observed for As, but with mean Igeo values falling in the class of moderately contaminated-heavily contaminated class in most of the estuary.

### Enrichment factor (EF)

Table 7 also shows the EF values calculated using Fe as normalizer (Zhang and Liu 2002). Where the index is >1.5, the source of the element is due to anthropic pollution and not to crustal materials or natural weathering processes. This is the case in decreasing order of Ni, Cd, As, Pb, U and Hg. EF values of Ni were very high, in the range of 9.89–12.37, with an increasing trend oriented to the estuary water where the recorded peak was up to ~10 fold 1.5, while in the case of As, Cd and Pb pollution which was rather similar at all sites and over the three seasons, Ni reached the highest peaks during the monsoon and at all sites. Multiple sources of Ni pollution could be identified like metallurgical activity, petroleum refineries, intensive activities of the bay shipyards of the HRE where vessels are maintained and repaired and the intense use of antifouling paints (Nemr et al. 2006; Costa et al. 2016). This finding seems to match with those from a study on the HRE water (Mukherjee et al. 2015): authors revealed that Ni and Cd significantly accumulated in the most consumed fishery resources of Hooghly area, *Mystus cavasius* and *Glossogobius giuris* posing a great risk for public health since elements go beyond the permissible level.

### Contamination factor (Cf), modified contamination factor (mCD)

The contamination factor Cf of each element, Table 8, was used to evaluate the contamination of the single trace element (Jiang et al. 2013; Kerolli-Mustafa et al. 2015). The contamination levels were classified based on their intensities on a scale, ranging from 1 to 6 (Hakanson 1980). Based on their mean Cf values and over 2014–2017, TEs ordered with the following sequence: Ni>Cd>As>Hg>Cr>Pb>U>Co>Cu>Fe>Mn>Zn>Al. Cf of Ni was > 6 and displayed higher peaks in the estuary water, up to 12.4; that of Cd was close to 6 especially during post-monsoon and decreases toward the outer estuary. Thus, both Ni and Cd represented a very high contamination situation, as was in the class of considerable contamination, whereas Co, Cr, Cu, Fe, Mn, Pb and U fall in the class of moderate contamination with Cf ranging between 1 and 3. From this index, two further indexes were calculated, the contamination degree, CD, which represents the sum of the contamination factors, and the modified contamination degree (mCD) calculated summing the single Cf values divided by the number of TEs. According to the classification of mCD proposed by Abrahim and Parker (2008) and to the mean values of mCD of ~2.50, the HRE was polluted at a moderate degree, 2< mCD < 4, with limited spatial and temporal differences.

### Pollution load index (PLI)

The pollution load index PLI according to Tomlinson et al. (1980) was also calculated. Sekabira et al. (2010) reported that PLI > 1 indicated anthropogenic inputs. Data reveal how the index was high at all sites with a mean HRE value of 1.41 and tendentially lower values, ~1.20, during the monsoon.

### Potential contamination index (Cp)

Finally, the potential contamination index, Cp, was calculated by the method of Hakanson (1980). Davaulter and Rognerud (2001) proposed low contamination if Cp < 1, moderate contamination 1 < Cp < 3 and severe or very severe contamination Cp > 3. Table 9 shows how most of TEs presented Cp values greater than 6, and hence, sediments were very severely contaminated with values for Cd, Ni and As of 9-, 6- and 6-fold the limit of the heavier polluted class, with worst situation for As and Cd at fresh and brackish water and during post-monsoon evidencing a rapid recharge of the pollution after monsoon and for Ni at estuary water during monsoon.

### Potential ecological risk index (Er)

In order to define the ecological risk in aquatic system, we calculated the potential ecological risk index, Er, as proposed by Hakanson (1980). The index serves to establish the degree of trace element pollution in sediments, according to the individual toxicity of TEs and the response of the environment.

### Comprehensive potential ecological risk index (RI)

From the Er index, it was calculated the comprehensive potential ecological risk index RI as the sum of all risk factors which was rated according to the method of Devanesan et al. (2017). *RI* represents the sensitivity of various biological communities to toxic substances and illustrates the potential ecological risk caused by TEs.

The Er of Cr, Cu, Pb and Zn, Table 10, was all below 40, placing these elements at low ecological risk level, the average Er of As and Ni classified these TEs at most sites and seasons at moderate risk level, whereas the Er of Cd falls in the higher-risk class with a peak of 284 at the freshwater site and in the post-monsoon season and a significant decreasing trend toward the outer part of the estuary. The *RI* values, Table 10, were clearly related to the degree of anthropogenic disturbance. The global risk is severe, 300≤RI<600, at all sites and seasons and especially after the monsoon, especially at fluvial and brackish locations where it is likely that intense anthropic discharge leads to increasing deposition of chemical elements. This scenario seems to link quite well with the recent study from NACER (2020) on the livelihood and health challenges faced by riverine communities of Ganga. The study found how fisher folk depends on river Ganga’s water for drinking and is likely to report higher incidences of diseases such as pneumonia, diarrhoea, cholera, cough/cold, fever, skin disease, typhoid and jaundice.

**Table 10:** Potential ecological risk factors, Er, and comprehensive ecological hazard index, RI

Er								RI
As	Cd	Cr	Cu	Hg	Ni	Pb	Zn	
Freshwater								
44.8	219	2.9	5.2	59	40	7.9	0.8	380
Brackish water								
38	190	3.4	5.5	58	46	8.1	0.8	351
Estuary water								
49	127	3.6	6.2	53	56	8.3	0.8	304

## Conclusions

The present study quantified and assessed the natural enrichment or anthropogenic sources, contamination levels and toxicity of TEs and U in sediment samples from the terminal stretch of the Hooghly river during the period 2014–2017. This study shows that the major sources of TEs contamination are land-based anthropogenic ones. It shows that the distribution and transportation of these elements in sediments are not uniform and the change in concentration is due to season alternance of dry and wet weather conditions besides man-made flows, water physico-chemical features, sedimentation and hydrodynamic conditions. Cf index shows how both Ni and Cd, even though with different spatial and seasonal behaviours, represented a very high contamination situation, whereas the Cp and PLI values revealed that sediments were very severely contaminated by most TEs. Based on the individual ecological risk, As, Cd and Ni are at moderate risk level, whereas global risk was severe closer to man-made inputs, where artisanal gold mining activities, agricultural runoff, lithology and other anthropogenic inputs are probable sources of TEs pollution. Results evidence the need for effective and efficient management policies to control TEs discharge into the estuary and detrimental effects on the nearby mangrove forest of Sundarbans.

## Data Availability

All data generated or analysed during this study are included in this published article (and its supplementary information files.
